# The Brain as a Distributed Intelligent Processing System: An EEG Study

**DOI:** 10.1371/journal.pone.0017355

**Published:** 2011-03-15

**Authors:** Armando Freitas da Rocha, Fábio Theoto Rocha, Eduardo Massad

**Affiliations:** 1 Research on Natural and Artificial Intelligence (RANI), Jundiai, São Paulo, Brazil; 2 School of Medicine, University of São Paulo, São Paulo, Brazil; University of Maribor, Slovenia

## Abstract

**Background:**

Various neuroimaging studies, both structural and functional, have provided support for the proposal that a distributed brain network is likely to be the neural basis of intelligence. The theory of Distributed Intelligent Processing Systems (DIPS), first developed in the field of Artificial Intelligence, was proposed to adequately model distributed neural intelligent processing. In addition, the *neural efficiency hypothesis* suggests that individuals with higher intelligence display more focused cortical activation during cognitive performance, resulting in lower total brain activation when compared with individuals who have lower intelligence. This may be understood as a property of the DIPS.

**Methodology and Principal Findings:**

In our study, a new EEG brain mapping technique, based on the *neural efficiency hypothesis* and the notion of the brain as a Distributed Intelligence Processing System, was used to investigate the correlations between IQ evaluated with WAIS (Whechsler Adult Intelligence Scale) and WISC (Wechsler Intelligence Scale for Children), and the brain activity associated with visual and verbal processing, in order to test the validity of a distributed neural basis for intelligence.

**Conclusion:**

The present results support these claims and the *neural efficiency hypothesis*.

## Introduction

Jung and Haier [1] reviewed studies from functional (i.e., functional magnetic resonance imaging and positron emission tomography) and structural (i.e., magnetic resonance spectroscopy, diffusion tensor imaging and voxel-based morphometry) neuroimaging paradigms and reported a striking consensus, suggesting that variations within a distributed network predict individual differences found in intelligence and reasoning tasks. They described this network in the Parieto-Frontal Integration Theory (P-FIT). The P-FIT model includes the dorsolateral prefrontal cortex (BAs 6, 9, 10, 45, 46, 47), the inferior (BAs 39, 40) and superior (BA 7) parietal lobule, the anterior cingulate (BA 32), and regions within the temporal (BAs 21, 37) and occipital (BAs 18, 19) lobes. White matter regions (the arcuate fasciculus) were also implicated. Various neuroimaging studies demonstrated that both frontal and posterior brain regions are associated with intelligence. As a result, it is now widely believed that a brain network characterized by interactions between multiple brain regions is likely to be the neural basis of intelligence [2].

The theory of Distributed Intelligent Processing Systems (DIPS) was first developed in the field of Artificial Intelligence to formalize systems comprised of multiple agents that have individual expertise in solving defined problems but gain the ability to solve tasks of greater complexity through cooperation. DIPS intelligence is, therefore, a function of the types of tools used by its agents, as well as how and for what purpose these tools are used [3]–[10]. Intelligence is both a function of agent diversity and the extent of versatility and plasticity of the relationships shared by these agents. Rocha et al have discussed at length the brain as DIPS [8], [11], [12].

DIPS reasoning is the cooperative activity among a collection of agents coupled, as much as possible, in a decentralized and loose manner that eventually provides a solution to a given problem. By loose, we mean that the relationship between the agents can easily be modified and can therefore account for a solution to a task. By decentralized, we mean that both control and data are logically and geographically distributed; neither global control nor global data storage exist. The control structure is not dependent on the knowledge and properties of specific agents (neurons). Instead, it is embedded in the rules that govern messaging among agents, or can be found in the chemical transactions at the synaptic level. Messages are exchanged by mail systems because each agent (neuron) knows how to address communication to, or has specific connections with, other specific agents (neurons) that may contribute to the task solution. Messages can also be exchanged by blackboard systems (e.g., working memory), where agents post information to be shared with or accessed by any other agents (neurons) that may contribute to a specific DIPS reasoning process (brain processing).

DIPS knowledge is distributed among its agents (neurons) according to their specialization, and is primarily encoded by the relationships (connections) shared by these neurons. In the case of memory, for instance, some agents (e.g., sensory neurons) are responsible for storing data (e.g., sensory information) while others (e.g., hippocampal neurons) keep track of the relationships between these pieces of data by storing information about the associations between these agents (e.g., connecting the different sensory neurons). In the case of procedural knowledge, some agents relate data (e.g., sensory or memorized information) to their processing tools (e.g., motor actions). The complexity of DIPS knowledge depends on the number of specialized agents (neurons) and the complexity of their relationships.

Task distribution is an interactive process between an agent with a task to be executed and a group of agents that may be contributing to task execution. Many of these agents may propose similar but not identical solutions to a given task, either because they may share information from different sources or because they use different tools to handle the same piece of information. This redundancy supports the robust degradation properties of DIPS because agents may be lost without greatly affecting the system's performance. However, this same redundancy may also cause conflict, which, in turn, requires task solutions to be carried out under the guidance of special rules implemented by specialized agents (for examples, see [4], [6], [11], [12]).

EEG mapping studies of the physiological correlates of human intelligence have focused on the level and topographical distribution of cortical activation. The experiments clearly showed that EEG recordings correlate with intellectual abilities [13]–[20]. In fact, strong empirical evidence suggests that individuals with higher intelligence display more focused cortical activation during cognitive performance, resulting in lower total brain activation compared with individuals who have lower intelligence. Such data support the *neural efficiency hypothesis* [19], [21]. Additionally, a high level of expertise was beneficial for good task performance, but exerted a topographically distributed influence on cortical activation patterns. These findings suggest that higher cognitive performance and the underlying cortical activation are not simply a function of knowledge and competency in a specific domain. They are also a function of the efficiency of information processing by widely distributed systems [16].

Rocha et al [9], [12] proposed that the brain is a DIPS formed by collections of loosely interacting neurons (agents) specialized for data collection (sensors), problem solving (associative neurons), data communication (interneuronal systems), acting upon the surrounding environment (motorneurons), etc. Based on the *neural efficiency* and DIPS hypothesis, the authors developed a new technique for EEG brain mapping, and applied it to the study of arithmetic cognition in children and adults. The rationality of such approach is presented in the Appendix S1. Principal component analysis showed three distinct patterns of neuronal recruitment for arithmetic calculations in all experimental groups, varying with the type of calculation, age and sex (figure 8).

The purpose of the present paper is a) to introduce a formal model of DIPS intelligence that may be useful for understanding human intelligence and its neuropsychological substrates; b) to use the above EEG mapping technology in order to investigate the correlations between IQ (evaluated with WAIS [Wechsler Adult Intelligence Scale] or WISC [Wechsler Intelligence Scale for Children]) and brain activity associated with visual (puzzle solving and mental rotation) and verbal processing (charade comprehension and text understanding); and c) test the validity of the proposed theoretical construct.

## Results

The mean adult IQ value was 103 and the mean child IQ value was 99, thus, intelligence was equivalent in the two experimental groups. There was no difference in IQ according to abilities of visual and verbal reasoning (Table 1). No statistical IQ or RT differences were observed between genders. RT was smaller for adults compared with the children, and the correlation coefficient for RT×A was −0.51 (R^2^ = 0.26).

**Table 1:** RT Factor analysis.

Factor Loadings (Unrotated)	
	Factor 1
Mr	0.862152
Pz	0.900775
Ch	0.738045
St	0.743899
Expl.Var	2.652798
Prp.Totl	0.6632
Eigen value	2.652798

Mr – Mental Rotation RT; Pz – Puzzle RT; Ch – Charade RT; St – story understanding RT, Expl. Var – explained variance; Prp.Totl – probability.

**Figure 1:**
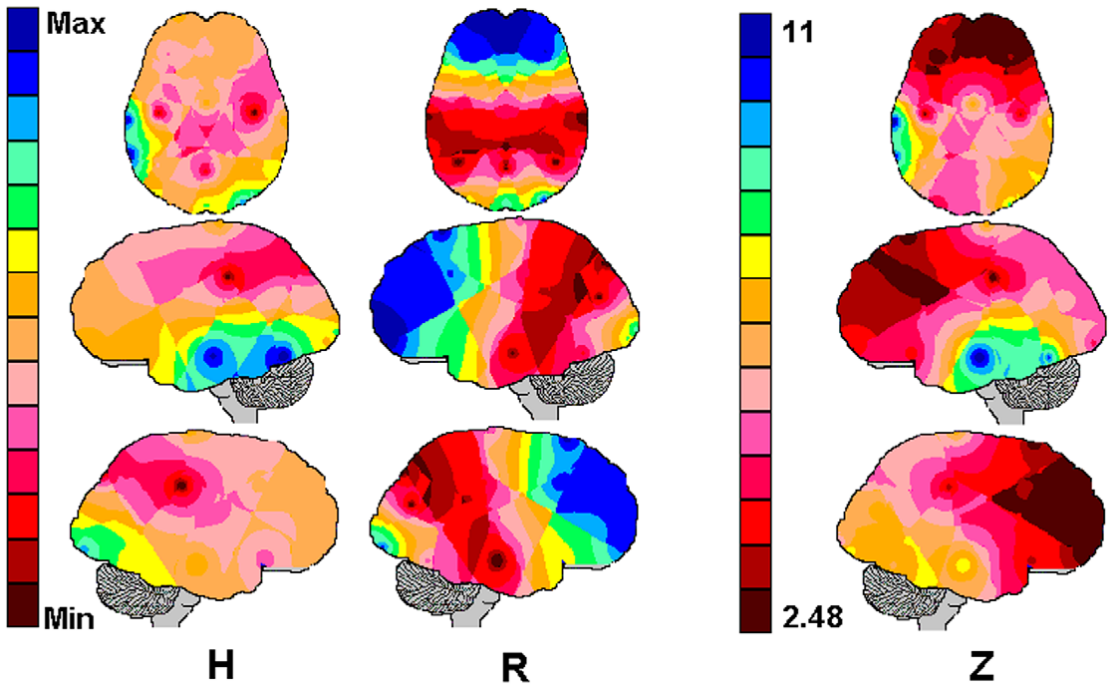
Comparison between a “Hypothetical” (H) and the Real (R) Brains. The Hypothetical brain was obtained by randomizing the calculated entropies (see text for further details. The actual calculated entropies were used to obtain the Real brain. The mappings H and R depict the averaging of these entropies. The comparison between averaging mappings H and R was quantified by the Z-scores shown by the mapping Z. Noted that the minimum Z-score was 2.48 and the maximum was 11.

**Figure 2:**
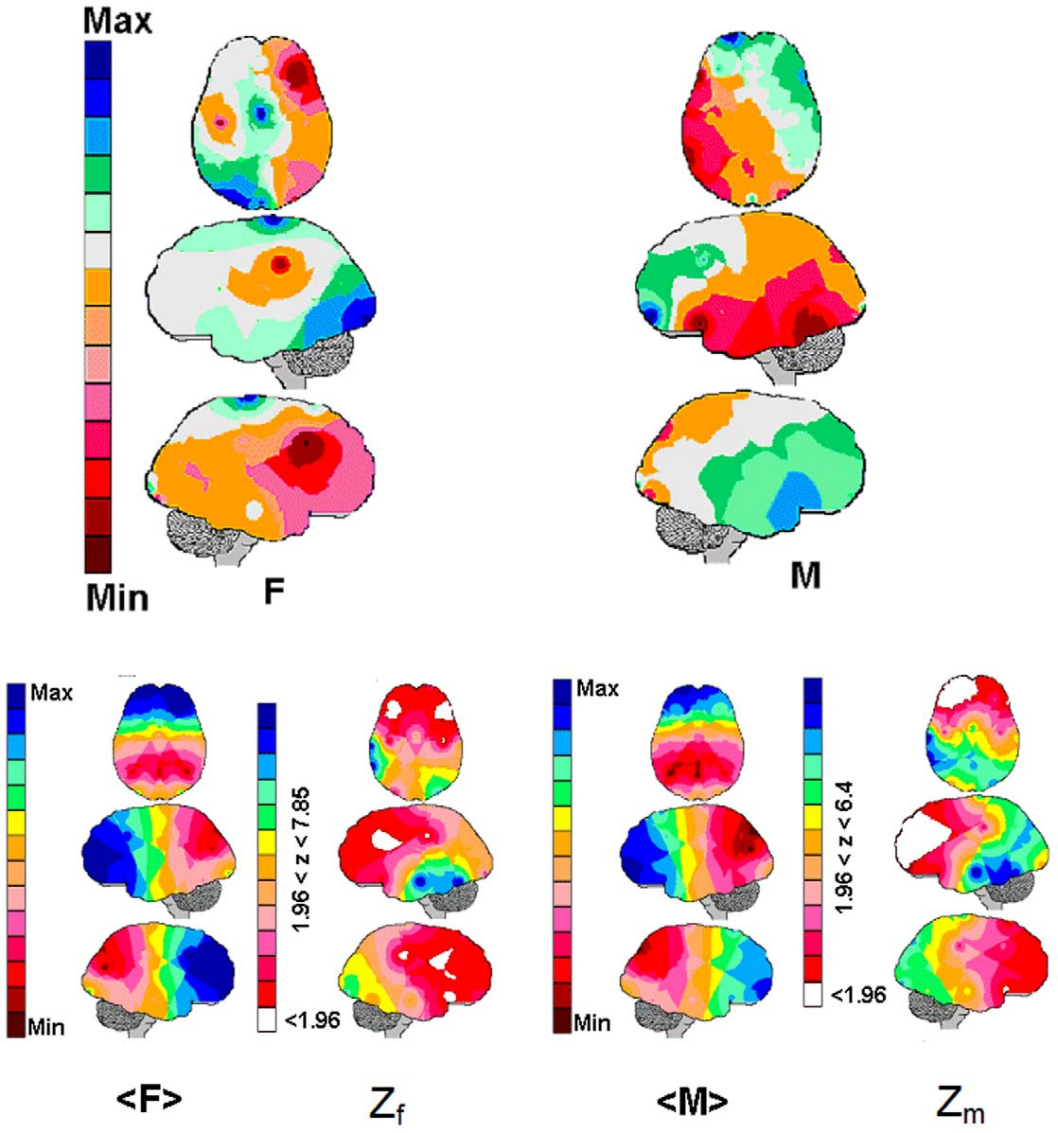
The EEG mappings for the calculated linear regression. 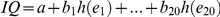
 calculated for females (F) and males (M) Legends as in Figure 1. The Pearson's correlation coefficient between the regression mappings F and M was −0.27. The Z mappings Z_f_ and Z_m_ mappings show the differences between the averaging mappings <F> and <M> and the Hypothetical brain H in figure 4. The areas for which the Z-score is smaller than 1.96 are shown in white, and the areas for the Z-score is greater than 1.96 are collored according to the magnitude of the Z-score.

**Figure 3:**
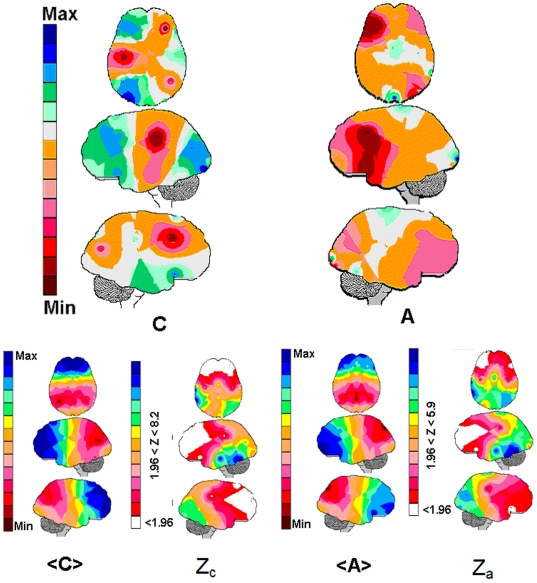
The EEG mappings for the calculated linear regression. 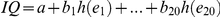
 calculated for adults and children Legends as in Figure 1. The Pearson's correlation coefficient calculated for these mappings was −0.16. The Z mappings Z_c_ and Z_a_ mappings show the differences between the averaging mappings <C> and <A> and the Hypothetical brain H in figure 4. The areas for which the Z-score is smaller than 1.96 are shown in white, and the areas for the Z-score is greater than 1.96 are collored according to the magnitude of the Z-score.

**Figure 4:**
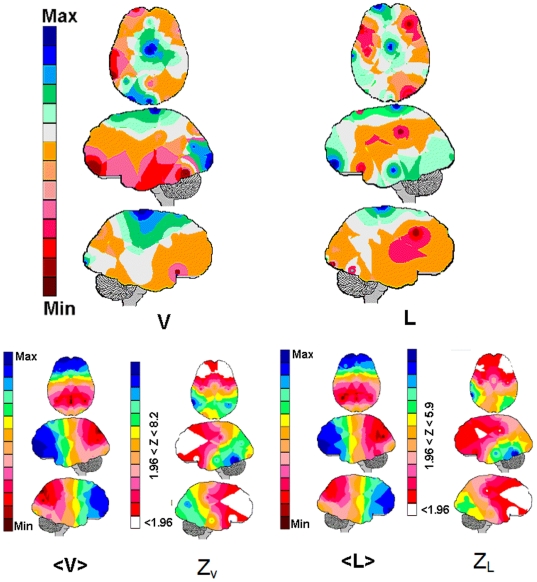
The EEG mappings for the calculated linear regression. 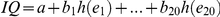
 calculated for visual (V) and verbal (L) games Legends as in Figure 1. The Pearson's correlation coefficient calculated for these mappings was 0.22. The Z mappings Z_v_ and Z_L_ mappings show the differences between the averaging mappings <v> and <z> and the Hypothetical brain H in figure 4. The areas for which the Z-score is smaller than 1.96 are shown in white, and the areas for the Z-score is greater than 1.96 are collored according to the magnitude of the Z-score.

The Z-scores results for the comparison between the Hypothetical Brain and the Real brains of the studied population are shown in figure 1 and they clearly demonstrate that entropy values associated with the different types of brain are significantly different, since the minimum Z-score obtained was 2.46. Therefore, our null hypothesis was rejected. These differences persisted throughout all other comparisons between the hypothetical brain and the brains differentiated by gender (figure 2), age (figure 3) and tasks (figure 4). For these comparisons, the Z-score obtained for some electrodes did not reached the 0.05 significance cutoff (shown as white areas in the figures). However, the number of such electrodes were always smaller than the electrodes that attained statistical significance.

Table 2 shows the calculated values for 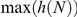, 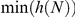, 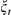 and 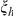 for each game. Mental Rotation (**Mr**) was associated with the smallest and Story Understanding (**St**) with the highest 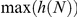. The value of 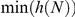 was almost the same for all games. The values of 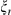 ranged from 0.27 in the case of the verbal games to 0.48 in the case of the puzzle. The value of 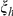 was around 0.7 for all games.

**Table 2:** The efficiency values.

	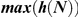	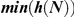	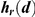	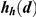	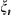	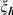
Mr	68	11	24	8	.35	.72
Pz	76	11	37	8.5	.48	.77
Ch	76	11	22	7.5	.27	.78
St	79	12	22	7.5	.27	.73

Mr - Mental rotation; Pz – Puzzle; Ch – Charade comprehension; St – Story understanding.

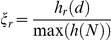
, 
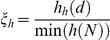
, 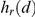 the entropy for random decision and 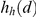 the entropy for heuristic decision.

The multiple regression analysis revealed two statistical models correlating IQ with age (A), RT, 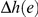 and 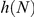 and with 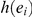. Model 1 positively correlated IQ with age (A) and 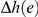, and negatively correlated IQ with RT and 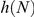. The regression coefficient R for this model was 0.54 and explained 0.29 of data variance. Model 2 positively correlated IQ with age (A) and 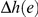, and negatively correlated IQ with RT. The regression coefficient R for this model was 0.48 and explained 0.24 of data variance.

**Figure 5:**
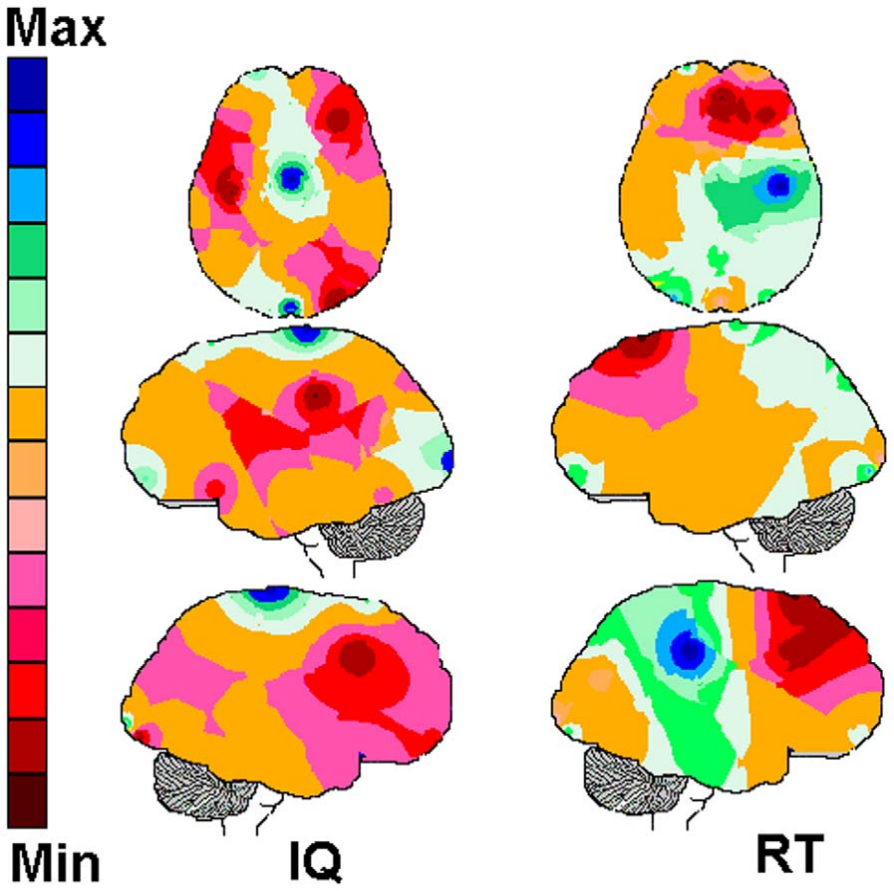
The EEG mappings for the calculated linear regressions. 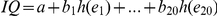
 and 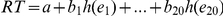 Light to dark blue areas are those for which 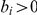 and pink to dark red areas are those for which 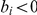. Max and Min – maximum and minimum values for 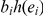 , respectively. The Pearson's correlation coefficient between IQ and RT mappings was −0.16.

The multiple regression 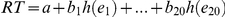 was used to generate the 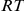 mapping in figure 5. 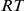 decreased as 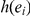 increased for the anterior (mainly central and right) electrodes and increased for the posterior (mainly central and right) electrodes.

The multiple regression 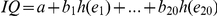 was used to generate the 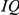 mapping in figure 5. The 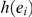 values calculated for FP1, FZ, CZ and OZ were directly related to IQ, implying that IQ increased as 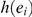 for these electrodes increased. In contrast, 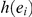 values obtained for C3, F4 and O2 were inversely related to IQ; thus, IQ decreased as 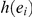 for these electrodes increased.

The multiple regression 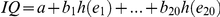, calculated separately for women and men, was used to obtain the brain mappings shown in figure 2. The Pearson's correlation coefficient for these two mappings was −0.27. In women, high 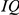 is mostly associated with high 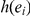 for the left brain and with low 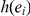 for the right hemisphere electrodes. In men, an opposite pattern was observed, with high 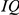 mostly associated with high 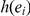 for the right brain and low 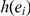 for the left hemisphere electrodes.

The multiple regression 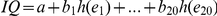, calculated separately for adults and children, was used to obtain the brain mappings shown in figure 3. The Pearson's correlation coefficient for these two mappings was −0.16. The inverse correlation between 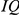 and 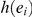 calculated for the right anterior frontal and right posterior electrodes, was similar in both groups. The main difference between the two mappings was the opposite relationship between 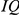 and 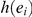 calculated for the left anterior electrodes. For these electrodes 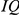 was inversely correlated with 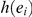 in adults and directly correlated with 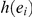 in children. Finally, a positive correlation between 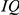 and 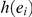 generally dominated in children compared with adults.

The multiple regression 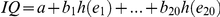, calculated for the visual and verbal games, was used to obtain the corresponding brain mappings shown in figure 5. The Pearson's correlation coefficient for these two mappings was 0.22. The direct correlation between 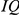 and 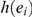 calculated for the central (FZ, CZ and OZ) and right posterior (P3 and O1) electrodes, was similar in both groups. An inverse correlation of 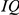 with 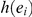 was observed for the F4, F7, C3 and O2 electrodes in verbal games. In contrast, the opposite relationship was seen for the FP1, T3 and T5 electrodes when verbal and visual games were compared.

## Discussion

In the field of physiological study of human intelligence, there is strong evidence of a more efficient operation (i.e., less activation) of the brain in brighter individuals (the *neural efficiency hypothesis*). Haier et al. [22] observed a negative correlation between intelligence and the extent of energy consumption (glucose metabolism) in the brain during cognitive task performance. These initial findings led the authors to formulate the *neural efficiency hypothesis of intelligence*, claiming that “subjects performing a complex task may well use a limited number of brain circuits and/or fewer neurons, thus requiring minimal glucose use, while poor performers use more circuits and/or neurons, some of which are inessential or detrimental to task performance, and this is reflected in higher overall brain glucose metabolism”.

Here, because our null hypothesis was rejected (see figure 1), the correlation coefficient r_i,j_ between the EEG activity recorded by the electrodes 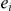, 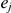 was used to calculate (equations 1 to 4) the entropy 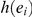, quantifying the commitment of the neurons recorded by the electrode 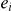 to solving a task 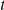 . The recruitment 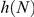 of the brain in solving the game 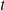 (equation 5) was set depending on the number 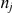 and 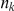 of neurons 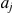 for which 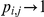 and 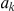 for which 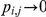, respectively (see figure 2 and equation 4). Finally, the brain efficiency in handling the task 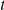 was defined as 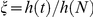 (equation 6), where 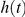 is the entropy of 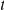.

In this context, the *neural efficiency hypothesis* implies 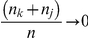 and 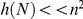, in order to make 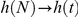 and 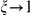. In other words, the *neural efficiency hypothesis* requires the number 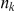 of neurons recruited for the task solution, and the number 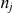 of neurons forbidden from participating in the task solution, to be as small as possible. Here, 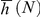 was statistically similar for the visual (40 bits) and verbal (43 bits) games (43 bits). Therefore, 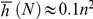 (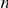 = number of recording electrodes = 20). In other words, the brain recruitment as measured by 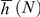 is equal to only 10% of the maximum entropy that could be measured by the 20 recording electrodes.

In addition, the calculated 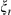 was smaller than 0.5 and 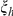 was greater than 0.7 for all games. The neural efficiency 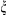 has to be greater than 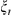 if random strategies were used to solve the games, and greater than 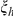 when heuristics were used to solve the tasks because 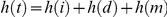. It may be proposed, therefore, that subjects solved the games with a *neural efficiency* 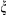 that was at least greater than 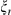, and thus greater than 0.5. It may also be hypothesized that 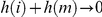 and 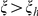, and therefore 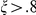, when individual knowledge increases and allows the subjects to create heuristics for efficient task solutions. In any of these cases, 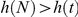 because 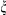 cannot be greater than 1. Recall, however, that 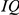 was inversely related to 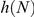. Therefore, heuristic solutions must predominate for high 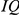, in order to decrease 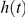 and keep 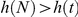. Similarly, a random solution may be used in the case of low 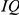 because 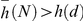 for all games. As a consequence, it may be concluded that 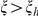 for high 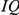 and, at least 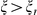 for low 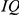.

Although the present results seem to confirm the *neural efficiency hypothesis*, caution is necessary because, as discussed above (see methods), the expected values of 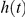 were obtained under the assumption that subjects used some optimizing strategies. In the case of the puzzle, the analysis of the sequences of piece placements showed that they used the proposed strategy of organizing the puzzle pieces into meaningful items in order to solve the game. However, there is no available information about the kind of possible heuristic used to solve the other games. In order to validate the present findings, future studies require specially designed games that allow the analysis of strategies used for their Solutions.

Graph theory allows the definition of what should be considered an optimal network. The notion of an optimal network is closely associated with the small-world phenomenon [23], [24]. The so-called small-world network architecture is distinguished from either ordered or random networks. On average, a sparsely connected graph is expected to have a lower clustering coefficient and longer path length compared with a densely connected graph with the same topology. Networks with small-world architecture are characterized by a combination of strong local clustering and a short characteristic path length (an index of global integration). This means that, although most of the connectivity is local, the network remains highly integrated due to a small number of long distance connections. Networks with scale-free architecture [25] are characterized by the presence of nodes with a very large number of long distance connections (the hub nodes). The likelihood 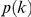 of a node having 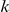 connections is given by 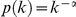, 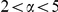. Broad-scale networks are characterized by a degree distribution that has a power law regime followed by a sharp cutoff that restricts the increase of 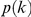. The cutoff function constrains the maximum number of nodes that may connect to hub nodes [26]. For example, 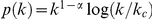 is a “broad-scale” network, where 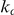 is the limiting degree. From equation 4, broad-range and scale-free networks have clusters of well-connected nodes 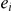 and 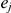 for which 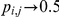 because the characteristic pathway length 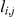 tends to 2 and a small number of hub nodes 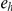 for which 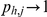 if 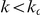.

Micheloyannis et al [27] recorded EEG signals to study neuronal interactions during working memory tests in individuals who had few years of formal education (LE) compared with individuals who had university degrees (UE). They quantified the synchronization between EEG channels in several frequency bands, and then converted EEG signal correlations into graphs to estimate the clustering and distance characteristics of the underlying processing networks. According to the authors, findings supported the *neural efficiency hypothesis* and suggested that the connections between brain areas of well-educated subjects engaged in working memory tasks have less small-world characteristics than those of less-educated volunteers. Iturria et al. [26] used diffusion-weighted Magnetic Resonance Imaging (DW-MRI) to estimated the anatomical connection probabilities (ACP) between 90 cortical and subcortical brain gray matter areas. They concluded that all the studied networks have small-world and broad-scale characteristics. Van den Heuvel et al [28] used a voxel-wise approach for a model-free examination of both inter-regional as well as intra-regional connectivity in the human brain. Resting-state 3 Tesla fMRI scans of 28 healthy subjects were acquired and individual connectivity graphs were formed out of all cortical and sub-cortical voxels with connections reflecting inter-voxel functional connectivity. Graph characteristics from these connectivity networks were computed. The clustering-coefficient of these networks turned out to be much higher than the clustering-coefficient of comparable random graphs. This result, together with a short average path length, indicated a small-world organization. Furthermore, the connectivity distribution of the number of inter-voxel connections followed a power-law scaling with an exponent close to 2, suggesting a scale-free network topology. Their findings suggested a combined small-world and scale-free organization of the functionally connected human brain. The results were interpreted as evidence for a highly efficient organization of the functionally connected brain, in which voxels are mostly connected with their direct neighbors, forming clustered sub-networks that are held together by a small number of highly connected hub-voxels that ensure a high level of overall connectivity.

The correlation coefficients 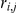 calculated for the EEG activity recorded by the electrode entropy 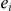 and 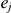 were assumed here to be surrogates for the connectivity between the neurons recorded by these electrodes. In this context, 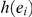 calculated for the recording electrode 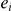 may be assumed to represent the connectivity 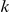 of the neurons recorded by 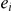. In other words, the number of instances 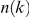, when the calculated 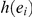 is equal to 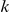, is a measure of 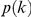 in the studied population. The regression analysis showed that 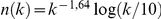, 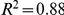 and 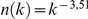, 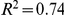, leading to the conclusion that the solution of our games was supported by broad-scale or scale-free networks.

In addition, the present results showed that 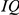 was inversely related to 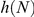 and directly related to 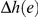. This means that high-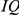 individuals tended to recruit fewer (smaller 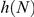) highly correlated (larger 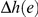) neurons, compared with low-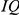 volunteers, to solve the games. Therefore, it may be proposed here that IQ is correlated with the dynamics of broad-scale (or scale-free) networks organized in the brain for different purposes (e.g., [29]–[31]). The information flow in this type of network is very efficient because it depends on a small number of connections (axons) with the hub nodes, instead of relying on a large number of randomly distributed connections, as is the case in random networks (e.g., [23], [26]).

The 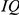 mapping in figure 5 shows that 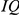 increased as 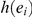, calculated for FP1 and the central electrodes (FZ, CZ and OZ), increased, and decreased as 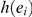, calculated for C3, F4, PZ and O2, increased. Lee et al [32] showed that high g-loaded tasks specifically increased regional activity in the bilateral fronto-parietal network that included the lateral prefrontal, anterior cingulate and posterior parietal cortices. In addition, the regional activations of the superior-g group were significantly stronger than those of the control group, especially in the posterior parietal cortex. Finally, regression analysis revealed that activity of the superior and intraparietal cortices (BA 7/40) strongly covaried with individual differences in g. Although EEG recorded activity cannot be easily mapped to spatial location, there is an almost perfect match between the 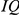 mapping in figure 5 and the map shown in figure 2 of Lee et al [32].

Haier et al [33] showed that more gray matter in a number of Brodmann areas (BA) was associated with higher 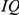, and suggested a distributed neural basis of intelligence similar to that disclosed by our data. Recently, Jung and Haier [1] reported a striking consensus among different papers suggesting that variations in a distributed network predict individual differences found in intelligence and reasoning tasks. They described this network as the Parieto-Frontal Integration Theory (P-FIT). The P-FIT model includes the dorsolateral prefrontal cortex (BAs 6, 9, 10, 45, 46, 47), the inferior (BAs 39, 40) and superior (BA 7) parietal lobule, the anterior cingulate (BA 32), and regions within the temporal (BAs 21, 37) and occipital (BAs 18, 19) lobes. The P-FIT model includes, therefore, many of the components presently identified by the 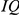 mapping in figure 5.

Another experimental approach to elucidating basic cognitive mechanisms that underlie general intelligence (psychometric g) is based on the attempt to relate psychometric g to the speed of information processing [34]. Within this conceptual framework, a large number of studies provided evidence for a relationship between levels of psychometric g and certain parameters of reaction time (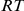) derived from Hick's law (e.g., [35]). In the present study, 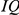 and 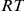 were also inversely related.

The 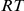 mapping in figure 5 shows that RT increased as 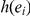, calculated for the posterior right hemisphere, increased, whereas 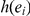 obtained from the right anterior electrodes decreased. There is some overlap between the 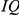 and 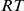 mappings (see figure 5); however, the activity recorded by FZ, P4 and O2 was related to 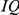 and 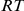 in opposite ways. The Pearson's correlation coefficient for the 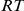 and 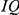 mappings was −0.16. These findings show that 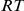 and 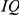 may share some common neural substrates but are two different neural constructs.

The consensus view states that there are no sex differences in intelligence. However, Lynn [36] has formulated a developmental theory of sex differences in intelligence that challenges this view. The theory states that boys and girls mature at different rates, such that the growth of girls accelerates at the age of about 9 years and remains in advance of boys until 14–15 years. At 15–16 years the growth of girls decelerates relative to boys. From this age on, boys continue to grow and increase their mean IQ relative to that of girls. Colom and Lynn [37] presented new evidence for the theory from the Spanish standardization sample of the fifth edition of the Differential Aptitude Test (DAT). Their results showed that sex differences for 18 year olds in the DAT performance as a whole is a 4.3 IQ point advantage for boys, a value that is very close to the advantage that can be predicted from their larger brain size (4.4 IQ points). Jackson and Rushton [38] found that 17- to 18-year old males averaged 3.63 IQ points higher than did their female counterparts on the 1991 Scholastic Assessment Test (SAT). Here, no IQ sex difference was observed, although it must be noted that the experimental group was small and involved both children and adults. Because of the small number of individuals in each experimental subgroup (n = 20) no gender statistics was separately analyzed for each of these subgroups.

**Figure 6:**
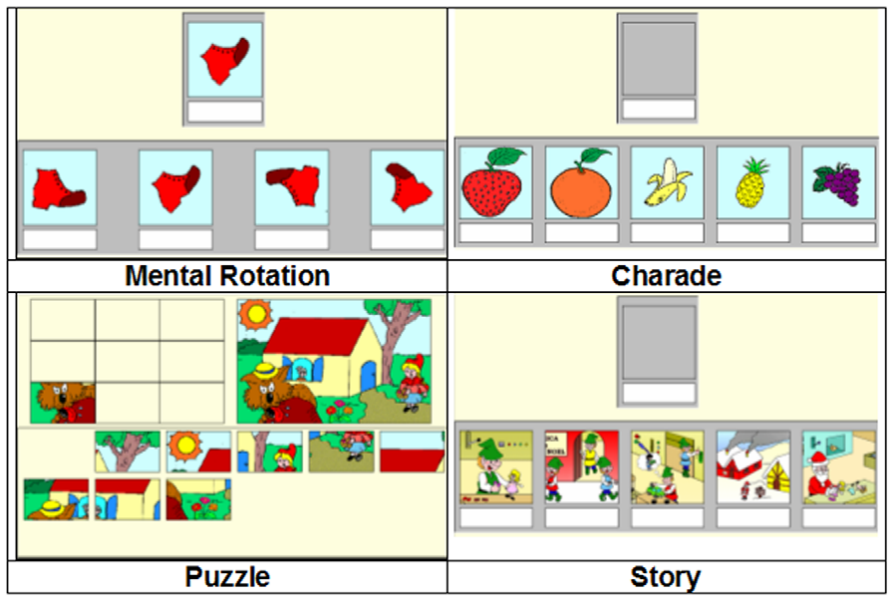
The games. The game rules are: a) Mental rotation (**Mr**): an object in a given spatial orientation is provided as a model to be matched to one out of four possible spatial orientations of the same object. Each game session involved 10 decision-making trials designed to explore visual reasoning; b) Puzzle solving (**Pz**): nine pieces of a scene, animal or object must be assembled over a nine-cell rectangle. The entire game includes three different pictures to be assembled. A warning signal indicates any piece misplacement. In this case, the subject had to remove the misplaced piece before trying another piece. Each game session involves at least 27 trials of decision-making designed to explore visual planning and reasoning; c) Charade solving (**Ch**): a three to four phrase description of a fruit or animal (e.g.; “My juice is delicious, my colour is my name; …”) is provided 500 ms before different pictures are displayed for decision making. Mean soundtrack duration for all charades is around 4 seconds. Each game session involved 10 trials of decision-making designed to explore speech comprehension and semantic memory; d) Story understanding (**St**): a verbal description of scenes of a Christmas story is provided 500 ms before five Christmas scenes are visually displayed for decision-making. Mean soundtrack duration for all scene descriptions is 5 seconds. Each game session involved 10 trials of decision making designed to explore speech comprehension and episodic memory.

Despite the fact that no 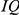 statistical difference was observed here between male and females, the 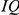 mappings calculated for male and females separately (see figure 6) showed interesting differences. The Pearson's correlation coefficient for the gender mappings is −0.27, the highest discrimination between all the mappings in figures 2, 3, 4, and 5. The right hemisphere and the anterior pole of the left frontal lobe are associated with high 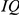 in males, whereas almost the entire left hemisphere correlated positively with the female 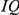 (green and blue areas in figure 2). In contrast, the left hemisphere (except O2) was negatively related with male 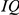, whereas female 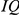 decreased as 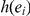 increased for the right electrodes F4, F8, P4 and O2 (red and pink areas in figure 2). Njemanze [39] evaluated cerebral lateralization during Raven Progressive Matrices in female and male subjects. Bilateral simultaneous transcranial Doppler (TCD) ultrasound was used to measure mean blood flow velocities (MBFV) in the right and left middle cerebral arteries (MCAs) in 24 (15 females and 9 males) right-handed normal subjects. The authors found that female subjects used a left hemisphere strategy, whereas males used a right hemisphere strategy to successfully solve Raven Progressive Matrices. According to the author, these results imply that intelligence is associated with neural systems within one hemisphere that are gender-accessible to a variety of cognitive functions. Neubauer et al. [19] found that in males, the highest correlations were observed for spatial 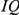, and in females for verbal 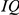. Furthermore, the sexes displayed topographical differences in neural efficiency patterns. Jaušovec and Jaušovec [18] described gender EEG differences concerning both general and emotional intelligence. Rocha et al. [9] used the presently discussed EEG brain mapping technology to study arithmetic cognition in children and adults. Factor analysis showed three distinct patterns of neuronal recruitment for arithmetic calculations in all experimental groups, which varied according to the type of calculation, age and sex. Males were faster in arithmetic calculation than females, irrespective of age. However, individuals of both sexes were equally accurate in their calculations. It is plausible to conclude that males and females have different cognitive styles that nevertheless result in no or minimal 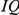 or cognitive differences.

Event-related brain potential (ERP) components showed typical gradual decrements in latency and amplitude with increasing age [40]. Regression analyses between Raven's intelligence scores and latency of the ERP components showed negative correlations for the late endogenous components at age 9. At ages 10 and 11, the earlier components showed positive correlations while the later components continued to show negative correlations. The amplitude measures showed only positive correlations, which shifted from the exogenous P1 component at age 9 toward the later endogenous components at ages 10 and 11. Here, the Pearson's correlation coefficient for the 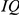 mappings calculated separately for children and adults (figure 3) was −0.16. The general picture revealed by these mappings seems to be that the children's networks involved in game solutions were broader than those used by adults. Fair et al. [41], [42] analyzed the connectivity of control networks and showed that adults, compared with children, used control networks with fewer short-range connections and more long-range connections. The authors concluded, as we did, that adult networks are more cohesive and interconnected than the corresponding children's networks.

In conclusion, we proposed a general prediction model that assumes IQ to be linearly correlated with 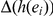, age (A), response time (RT), 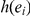 or 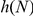. This model relates IQ to: the adequacy 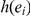 of the recruitment of neurons for solving a given task 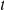 of complexity 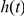; the extent 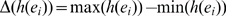 of this recruitment andits neural efficiency measured as 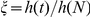, showing that these relationships are influenced by age and correlated with RT. The IQ and RT correlations with 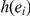 indicate that IQ and RT are better understood as different but correlated neural constructs.

Considering 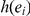 to be a measure of the connectivity 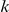 of the neural networks involved in our game solution, it was shown that these networks have broad-scale and scale-free properties. Broad-scale and scale-free properties are assumed to belong to efficient networks from the point of view of information flow. Consequently, we proposed that IQ is associated with the broad-scale and scale-free qualities of the neural networks that support reasoning and cognition.

The network-based understanding of brain function is a very recent paradigm that is being tested by neuroscientists as a formal tool to model cerebral function. Here, we used such an approach to study the relationship between IQ and the brain. The results are promising; however, much more work remains to account for some of the weak points of the present investigation and to research complex issues such as IQ, sex and age.

## Materials and Methods

### The experiment

Volunteers of two experimental groups

- Children (**C**): 20 children of both sexes (10 female and 10 male), age ranging from 7 to 11 years (mean = 9.15; sd = 1.38), who were attending an elementary school program, and
- Adults (**A**): 20 adults of both sexes (10 female and 10 male), age ranging from 23 to 45 years (mean = 29.2; sd = 5.85), all having finished college

played four different types of computer games (figure 2) while their EEG was recorded (20 electrodes placed according to the 10/20 system; impedance smaller than 10 Kohm; notch filter 50 Hz; sampling rate of 256 Hz and 10 bit resolution). The subjects' IQ was evaluated by the Wechsler Intelligence Scale for Children (WISC), or the Wechsler Adults Intelligence Scale for Children (WAIS) in a different session.

### The games

Four different types of games were used (figure 6):

- Puzzle solving (**Pz**): nine pieces of a scene must be assembled over a nine-cell rectangle to match the template figure.
- Mental rotation (**Mr**): the task is to mach the actual spatial orientation of a target object to one out of four different spatial orientations.
- Charade solving (**Ch**): a verbal description of four attributes of a fruit or animal is provided 500 ms before five different pictures of fruits or animals are displayed. The task is to match the verbal description to one out of five of these pictures.
- Story understanding (**St**): a verbal description of four attributes of Christmas story scenes is provided 500 ms before Christmas scenes are visually displayed 500 ms before five different pictures of the story are displayed. The task is to match the verbal description to one out of these five scenes.

Factorial analysis showed that **RT** for all games heavily dependent on one general factor (Table 3).

**Table 3:** The mean response time for each game.

	RT	SD
Mr	4.07	1.01
Pz	7.63	2.34
Ch	5.36	1.51
St	10.49	3.32

Mr - Mental rotation; Pz – Puzzle; Ch – Charade comprehension; St – Story understanding.

The calculation of the efficiency 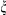 in solving each game requires the estimation of its 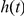 (see equation 6). The game solution implies at least three main steps:

1. identification: recognizing the objects, scenes and puzzle pieces as well as the meaning of each phrase that describes their attributes;
2. decision-making: selecting the assumed game solution, and
3. mouse control: reporting the game solution

These steps require specific computational capabilities estimated as 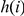, 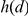 and 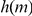, respectively. Therefore:
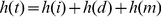
(1)The estimation of 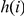 and 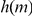 is difficult, but the possible range of 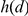 variation may be estimated as proposed below and provides some information about the lower limit of 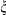 because 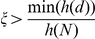. Also, 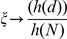 if 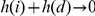 because both visual and/or verbal decoding, as well as motor control, are well learned.

The actual value of 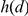 depends on the strategy used to solve the game. For example, the puzzle solution implies nine decisions about the relation of each puzzle piece and its location in the puzzle. If errors are made the number of decisions increases. A brute-force or random puzzle solution implies that the uncertainty 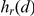 of this random decision is
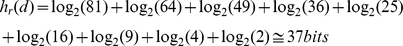
because:

- first decision implies that each piece location has the same probability of 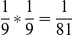;
- second decision implies the same probability of 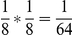 because if no error occurred in a), one piece of the puzzle was already located,
- and so on …

An alternative strategy to reduce the task entropy 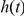 for puzzle solution, is to identify and organize the puzzle pieces into meaningful items. In the case of the puzzle in figure 6, these meaningful items are: the house composed of seven pieces; the wolf composed of three pieces; the tree composed of two pieces; the girl composed of two pieces; the sun represented by one piece and the flowers represented by one piece. If a subject decides first to locate the wolf pieces and then the house pieces, the uncertainty 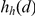 of this heuristic decision is:

- wolf: 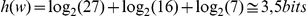 because the probability of its pieces location are respectively 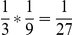, 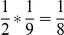 and 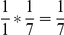 (as in a, b and c above) and
- house: 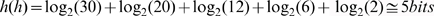 because the probabilities of the location of its pieces are respectively 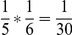, , 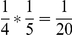, 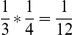, 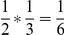 and 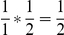 because two of the wolf's pieces are also house's pieces, and

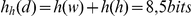
.

In the case of the rotation game, the total of all object orientations to be discriminated is eight. A brute-force solution implies 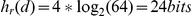 because the volunteer has to match (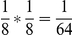) the orientation of the target and the orientation of the four possible solutions. Again, heuristics may be used to select meaningful general orientations like left, right, up, down, etc. in order to reduce the task entropy 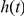. For instance, in the example in figure 2, a decision can be achieved in two steps: 1) the up/down (u/d) decision that implies 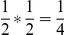 and 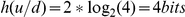, and then 2) the left/right (l/r) decision that implies 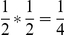 and 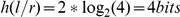, such that 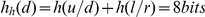.

In the cases of the charade and story games, each of the four verbal descriptions has to be matched to each of their visual counterparts in five different pictures; that is, each description has to be matched against 20 visual alternatives. A brute-force solution implies 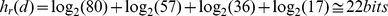 because the volunteer has four decision steps represented by the probabilities 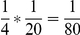, 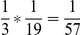, 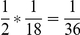 and 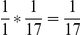. However, because the number of attributes necessary to solve many of the tests is smaller than four, the volunteer may use different strategies to reduce 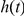. The reduction to three discriminating factors decreases 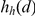 to 14 bits, whereas the reduction to two discriminating factors decreases 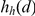 to 7.5 bits.

### The EEG recording and processing

**Figure 7:**
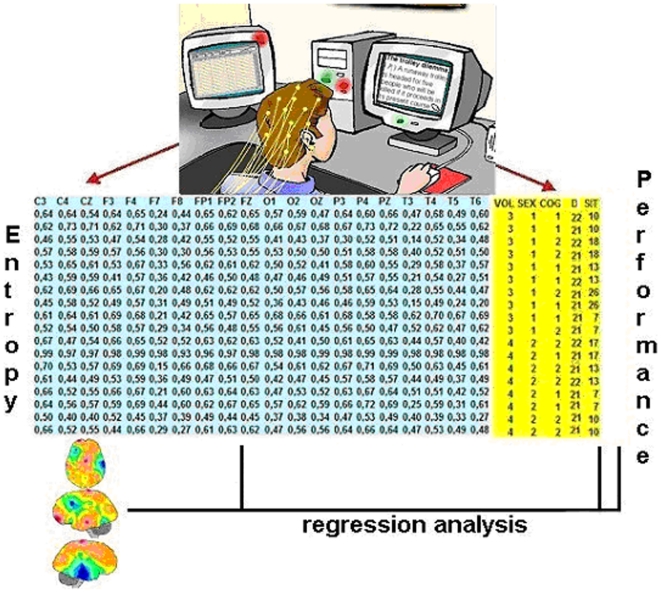
The experiment. Two networked microcomputers were used to record the EEG activity (10/20 system) while the individual is solving a specific cognitive task. The beginning of each task and the moment a decision is made are saved in the database together with the type of decision-making (D) and time required (response time ST) to achieve such decision. The linear correlation coefficients r_i,j_ for the recorded activity at each recording electrode e_i_, with reference to the recorded activity for each other 19 recording sites e_j_, were calculated for each game (COG) performed by a given subject. These r_i,j_ were used to calculate the correlation entropy h(e_i_) for each recording electrode e_i_. In this way, h(e_i_) was calculated for all 20 recoding electrodes. The corresponding values of h(e_i_) constitute the Entropy Data Base. Regression analysis between RT and h(e_i_) was used to build the cognitive mapping. Each mapping shows the contribution β_i_ h_m_(e_i_) of each electrode e_i_ to ST. h_m_(e_i_) is the average of h(e_i_) calculated for all subjects. IQs values of each subject were added to each corresponding data base record.

Two networked personal computers were used (figure 7): one to record the EEG and the other to display the game. Times for each test display (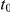) and decision (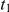) were recorded and synchronized with the EEG recording. Table 3 shows the mean 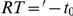 and 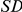 calculated for each game. After the experiment, EEG was visually inspected for artifacts that could compromise the analysis, and such records were discarded. Two EEG epochs of two seconds (from 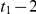 to 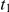, and from 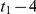 to 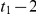) preceding the decision were selected to calculate the entropy 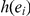 for each recording electrode 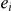 according to equations 1 to 4. We assumed the linear regression coefficient r_i,j_ calculated for the EEG activity recorded by the electrodes 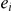, 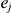 as the measure of the p_i,j_ of message exchange between the neurons recorded by these electrodes. Equation 6 was used to calculate 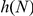.

**Figure 8:**
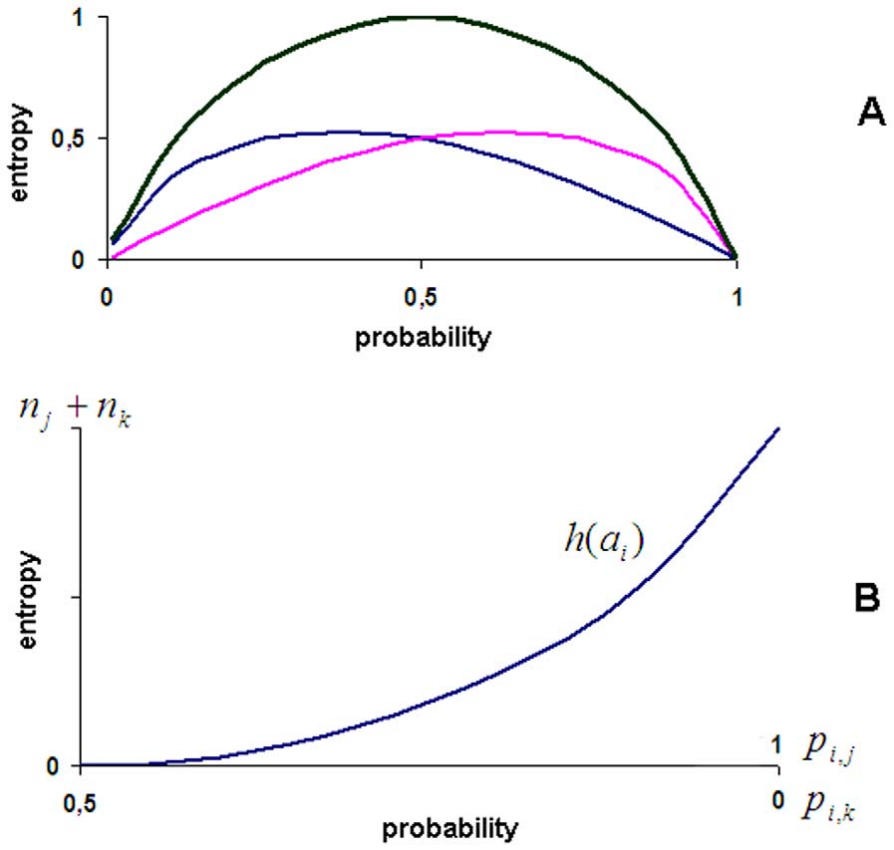
The message exchange entropies 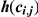 for agent 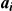 (A) and the entropy 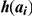 of the recruitment of the agent 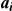 in the solution of a task 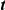 (B). See text for further explanation.

Here, we assumed that the null hypothesis states that the entropy calculated as above is equal to the brain activity of a hypothetical brain obtained by 1) randomly reordering the recorded EEG activity by the 20 channels and then 2) computing the entropy of a “hypothetical” brain using the randomized EEG activity. Any difference between this “hypothetical” and the real brains of the studied sample is quantified by calculating the Z scores between the hypothetical and these real brains.

The following efficiency coefficients were calculated:
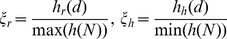
(2)Since 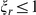 and 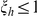 then:
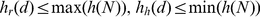
(3)Multiple regression analysis was used to calculate:
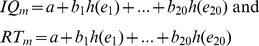
(4)as well as the significance 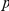-level 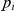 of each angular coefficient 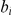. In addition, the number 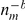 of negative angular coefficients with 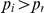 and the number 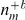 of positive angular coefficients with 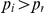 were obtained for a given significance level 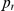. Finally, the values of 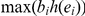, 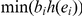 and 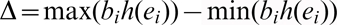 were obtained.

The normalized values of 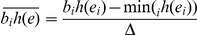 were used to build the brain mappings to display the results of the regression analysis (figures 2, 3, 4, and 5). The mapping color-encoding routine was obtained with commercial software (Icelera Inc.). Statistically positive 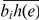 values (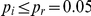) are encoded from red (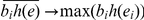) to yellow (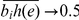); statistically negative 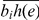 values (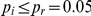) are displayed from blue (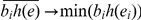) to green (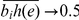), and statistically non-significant 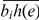 values (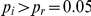) are shown in orange. Brain contours are used as references for spatial location of the 10/20 system electrodes. The Pearson's correlation coefficient was used to measure the similarity between regression mappings.

Multiple regression analysis was also used to study the correlations between IQ, RT, Sex, Group and Game, as well as to investigate a linear model of IQ depending on 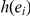, RT, Group and Game, and 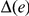 = Max(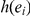)−Min(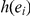). Statisca® was used for all statistical analysis.

### Ethical Aspects

This work was reviewed and approved by the Institutional Review Board of APAE-Jundiai (Associação de Pais e Amigos dos Excepcionais de Jundiai) and written consent was obtained from all participants in case of adults and from parents/guardians in the case of children.

## Supporting Information

Appendix S1 **Loosely connected DIPS.**
(DOC)
