# Supplementary material for: The Brain as a Distributed Intelligent Processing System: An EEG Study
Source: PLoS One. 2011 Mar 15;6(3):e17355. doi: 10.1371/journal.pone.0017355 (PMC3057967; doi:10.1371/journal.pone.0017355)
Supplement: Appendix S1 — Loosely connected DIPS. (DOC) [file pone.0017355.s001.doc]

# Appendix S1

**Loosely Connected Dips**

Let $N$ be a **DIPS** composed by $n$ agents$a_{i}$**,** each specialized according to the tools available to solve a given task. Also, let $p_{i,j}$ be the probability of message exchange between agents $a_{i}$,$a_{j}$. For a loosely connected **DIPS**, it is required that:

a) $p_{i,k}\to1$ for those $n_{k}$ agents that have suitable tools for the task solution;

b) $p_{i,j}\to0$ for those $n_{j}$agents having non-suitable tools for the task solution, and

c) $p_{i,l}\to0,5$for the remaining $n_{l}=n-(n_{k}+n_{j})$agents.

The mean probability $\mathop{p}\limits^{\_} _{i}$ of $a_{i}$ message exchange with any other agent $a_{j}$ belonging to $N$ is:

$\mathop{p}\limits^{\_} _{i}=\frac{\sum_{j=1}^{n} p_{i,j}}{n}$ (A1)

In this context, the entropy $h(c_{i,j})$of the message exchange between agents$a_{i}$,$a_{j}$ may be computed as proposed by Rocha et al. [9]:

$h(c_{i,j})=-p_{i,j}\log_{2}p_{i,j}-(1-p_{i,j})\log_{2}(1-p_{i,j})$ (A2)

such that (Figure 8):

d) $h(c_{i,j})\to0$ if $p_{i,j}\to1\text{ or 0}$ and

e) $h(c_{i,j})\to1$ if $p_{i,j}\to0.5$.

The adequacy of the enrollment of agent $a_{i}$ in the task solution depends on $\mathop{p}\limits^{\_} _{i}$ because $\mathop{p}\limits^{\_} _{i}\to1$ or $\mathop{p}\limits^{\_} _{i}\to0$implies that agent $a_{i}$ has a very broad or no specialization and therefore does not contribute to the task solution. The mean entropy $h(\mathop{c}\limits^{\_} _{i})$of agent $a_{i}$ message exchange is calculated as:

$h(\mathop{c}\limits^{\_} _{i})=-\mathop{p}\limits^{\_} _{i}\log_{2}\mathop{p}\limits^{\_} _{i}-(1-\mathop{p}\limits^{\_} _{i})\log_{2}(1-\mathop{p}\limits^{\_} _{i})$ (A3)

such that $h(\mathop{c}\limits^{\_} _{i})\to0$ if $\mathop{p}\limits^{\_} _{i}\to1\text{ or }0$ and$h(\mathop{c}\limits^{\_} _{i})\to1$ if$\mathop{p}\limits^{\_} _{i}\to0,5$.

Finally, the entropy $h(a_{i})$of the adequacy of $a_{i}$ enrollment in the task solution can be calculated (Rocha et al, 2005) as:

$h(a_{i})=\sum_{j=1}^{n} h(\mathop{c}\limits^{\_} _{i})-h(c_{i,j})$ (A4)

such that:

a) if $\frac{n_{k}+n_{j}}{n}\to0$ then $h(a_{i})\to n_{j}+n_{k}\leq n$

and

b) if $\frac{n_{k}+n_{j}}{n}\to1$ and$\frac{n_{j}}{n_{k}}\to1$ then $h(a_{i})\to n_{j}+n_{k}=n$

The constraints in a) set the minimum condition for $a_{i}$ enrollment in $t$solution as $n_{k}+n_{j}>\delta>0$ and those in b) establish the necessary condition for maximizing this enrollment by maximizing $h(a_{i})$ (figure 8). In addition, if $n_{k}+n_{j}\cong0$, the agent $a_{i}$ has no participation in the task solution and $p_{i,r}\to0,5$ for all of the $n$ agents$a_{r}$.

Because each agent $a_{j}$ recruited by $a_{i}$ may in turn enroll other agents $a_{m}$ for the solution of a given task $t$**,** the commitment $h(N)$ of $N$ to solve the task $t$is calculated as:

$h(N)=\sum_{i=1}^{n} h(a_{i})$ (A5)

Thus, if $h(t)$ is the entropy of the task $t$**,** the efficiency $\xi$ of $N$ in solving it is calculated as:

$\xi=\frac{h(t)}{h(N)}\leq1$ (A6)

because it is assumed that $N$ cannot solve tasks that have complexity ($h(t)$) greater than $h(N)$.

Furthermore, the solution of task $t$ is supposed to allow the enrollment of agents using different tools to achieve the same goal. The number of such agents is defined by the **DIPS** tool plasticity. In this context:

$\rho=\frac{n_{k}+n_{j}}{n}$ (A7)

measures the redundancy of $N$, supporting its robust degradation.

Robust degradation is a key issue for the understanding of **DIPS** intelligence. Increasing the number of agents that use similar tools ($\rho=\frac{n_{k}+n_{j}}{n}\to1$) to solve $t$ increases $h(N)$ and decreases $\xi$. Thus, if the number of redundant agents rises, the capability of $N$ to solve $t$ becomes more resistant to damage inflicted upon these agents. However, redundancy favors conflict because it raises the number of agents that may propose similar (but not the same) task solution. This, in turn, requires the enrollment of agents specialized for conflict solution, which makes the$t$solution difficult. Therefore, $\rho$ must be kept as small as possible in order for $N$ to efficiently solve the task [4, 6, 11, 12].

Choosing a suitable $\rho$ is also a key issue for DIPS intelligence. Neuroimaging and electrophysiological studies suggest that the anterior cingulate cortex (ACC) is involved in the cognitive control of response-related action and conflict management. An EEG frontocentral negativity, which probably originates from the ACC, is usually enhanced in conflict-trials that demand an unexpected response. The ACC has also been implicated by fMRI studies in conflict detection and manipulation in cognitive and moral judgment. In fact, the ACC is one of the components of a frontotempoparietal circuit involved in conflict detection and management [43-49].

Summarizing the main ideas introduced in this section, the efficiency $\xi$ of a **DIPS** in solving tasks is assumed to be dependent upon:

1. quantity and diversity of its agents;
2. adequacy and plasticity of the tools employed by its agents;
3. adequacy of its mail (axons) and blackboard (working memory) systems;
4. adequacy of its rules and agents for conflict management, and
5. plasticity of agent commitment that contributes to a better setting of $\rho$.
